# Childhood Epilepsy; Prognostic Factors in Predicting the Treatment Failure

**Published:** 2017

**Authors:** Mohammad Mehdi TAGHDIRI, Mahmoud OMIDBEIGI, Sina ASAADI, Eznollah AZARGASHB, Mohammad GHOFRANI

**Affiliations:** 1 Pediatric Neurology Research Center, Shahid Beheshti University of Medical Sciences, Tehran, Iran; 2 Pediatric Neurology Department, Mofid Children’s Hospital, Faculty of Medicine, Shahid Beheshti University of Medical Sciences, Tehran, Iran; 3 Medical Student, Faculty of Medicine, Shahid Beheshti University of Medical Sciences, Tehran, Iran; 4 General Practitioner, Functional Neurosurgery Research Center, Shahid Beheshti University of Medical Sciences, Tehran, Iran; 5 Department of Community Medicine, School of Medicine, Shahid Beheshti University of Medical Sciences, Tehran, Iran

**Keywords:** Childhood, Epilepsy, Prognosis, Treatment failure

## Abstract

**Objective:**

We aimed to find the prognostic factors to detect the patients who fail the treatment of epilepsy, in the early stages of the disease

**Materials &Methods:**

This study was done on the epileptic patients attending the Neurology Clinic of Mofid Children’s Hospital, Tehran, Iran from September 2013 to October 2014. After defining the criteria for exclusion and inclusion, the patients were divided to two groups based on responding to the medical treatment for their epilepsy and indices were recorded for all the patients to be used in the statistical analyses.

**Results:**

The patients’ age ranged from 1 to 15 yr. There was 188 patients with refractory seizure in group 1 (experimental group) and 178 patient with well controlled seizure in group 2(control group).There was a significant different between serum drug level in both groups and patients with refractory seizure group had a lower serum drug level than control group. In both groups tonic-clonic was the most common type of seizure. Also the prevalence of brain imaging Abnormalityand other neurologic disorders was significantly higher in patients with refractory seizure in compare with control group.

**Conclusion:**

Children with seizure who suffer from refractory epilepsy need more attention and exact observation by the medical staff.

## Introduction

Epilepsy is a neurological condition characterized by recurrent seizures without any known reason. “A large proportion of epilepsy begins in childhood and prevalence of epilepsy in children is approximately 3.5 to 7.2 per thousand children” (1). 

By the time being, analyzing distinctive aspects of epilepsy, including the risk of treatment failure and the risk of recurrent epilepsy remission is taken into consideration. Different types of seizures occur in children, and diagnosis is based on seizure type and its cause. The disease course varies from early remission after the onset of using the first drug to a permanent and uncontrollable seizure with multiple drug failures. 

Although, recuperation from seizures is more common in children than in adults, unfortunately, despite using various types of drugs, the first period of drug treatment fails in many cases and not all the children with seizures can be cured. Six to 41% of children with seizures do not respond to medical treatment due to refractory or uncontrolledepilepsy or drug resistance (2-7). Drug resistance is one of the major problems facing the treatment of epilepsy even with the new drugs and medical science progress. Treatment failure in childhood epilepsy has been left unexplained, of little evidence and the existing data have many challenging differences (8-10). 

With regard to disability and mortality caused by refractory epilepsy, it is a big area of interest to assess the predictive factors of refractory epilepsy and to prevent the possible inability. Early diagnosis of refractory seizures helps to start treatment, including surgery or other interventions (11-13).Finding these predictive factors is important because the failure of the first treatment seems to be a factor of poor prognosis in responding to antiepileptic drugs (14-15). 

In clinical practice, it is consequential for children to be diagnosed quickly and determined in terms of responding to medical treatment or failure. Predicting the group of children with high chance of responding to the medical treatment is important not only for the child and for the parents but also for the health system. Health system allows parents to make their choice about the allocation of appropriate treatment budget or if necessary, decide to use other treatment options (16- 20). 

Few studies are designed to identify factors predicting treatment failure in patients with epilepsy and in some cases, conflicting factors are presented as influential; unfortunately, none of them is approved as a definite predictor of treatment failure (21-24). 

So far, fewer studies are done to define uncontrolled epilepsy, drug resistance and the timing for refractory epilepsy to happen in children than in adults. There is also a little evidence on the cause of the high rate of treatment failure in childhood epilepsy. Even not a single definition of refractory childhood epilepsy is provided. Some research studies were conducted on refractory epilepsy, but many others considered limited factors and serum levels of the drug. Some studies had age limits, capable of restricting the results itself as well (25-28). 

In this study, we intended to study epilepsy in children from birth to the age of 15 yr and factors affecting the patients with refractory epilepsy, like antiepileptic drug levels are compared in the control and treatment failure groups.

## Materials & Methods

This study was done on the epileptic patients attending the Neurology Clinic of Mofid Children’s Hospital, Tehran, Iran from September 2013 to April 2015. The study divided patients to two groups. The first group included patients who had epilepsy and responded well to the medical treatment (control group), and the second group patients were suffering from refractory epilepsy (subject group). 

This study was approved by the Shahid Beheshti University of Medical Sciences Ethics Committee, and patients included in this study gave their informed consent to participate. 

**Exclusion and inclusion criteria**

Exclusion criteria for the study were as follows: Involving in any underlying kind of absorption gastrointestinal disease, having febrile seizure, kidney or liver diseases or taking other drugs that interfere with antiepileptic drug absorption rate or its serum levels. As an inclusion criterion after identifying and classifying patients according to the etiology of epilepsy, patients with idiopathicepilepsy (including simple seizure, atonic seizure, tonic-clonic seizure, absence seizure, complex seizure and tonic seizure) were chosen for the study. All clinical diagnosis was made by expert pediatric neurologist in our center. One group including patients received particular medical treatment and responded well to the treatment and the other group received the same treatment protocol but the seizure continued in accordance with the definition of refractory epilepsy. 

After defining the groups, serum levels of antiepileptic drugs were measured. In both groups, drugs were of the same brand of a company. Levels of drug consumption in patients were compared with statistical analysis between the control group and the examining group and the relationship between serum levels and risk of treatment failure is calculated. However, gender, age, family history of seizure, history of hospitalization in the neonatal period, age at onset, duration of epilepsy, seizure type, EEG pattern, disorders associated with anatomical imaging studies and any other underlying neurological disorders were recorded. Finally, according to the information obtained by the relationship between drug levels and other factors investigated, the risk of developing refractory seizures to the treatment and the need to increase the dose in this population were studied. 

Routine measurement of serum levels of drugs was done during the treatment course so that the study would not be costly and time-consuming. 

**Study design**

Three hundred ninety-nine patients were enrolled in this observational study and were followed up for at least 19 months up to 2 yr. Twenty-six of them were excluded because of drug revise in the study period. We identified refractory epilepsy as more than 1 seizure after>2 drug treatment for 18 months. Overall, 178 patients of refractory epilepsy (case) and 188 patients of well-controlled epilepsy conforming to eligibility criteria were enrolled. The serum level of the drugs was recorded to determine which individual had reached the therapeutic level. In both case and control groups, numbers of males (95/178: 53.4%) were higher than females (114/188: 60.06%). This is for prevalence of seizure that is more common in males. 

**Data analysis**

The SPSS software (version 19, SPSS Inc., Chicago, IL, USA) was used for statistical analysis. T-test and Chi-square test were used to evaluate which factors are more common in the refractory seizure group than control group. In this study P-value< P-value<0.05 was statically significant.

## Results

**Fig 1:**
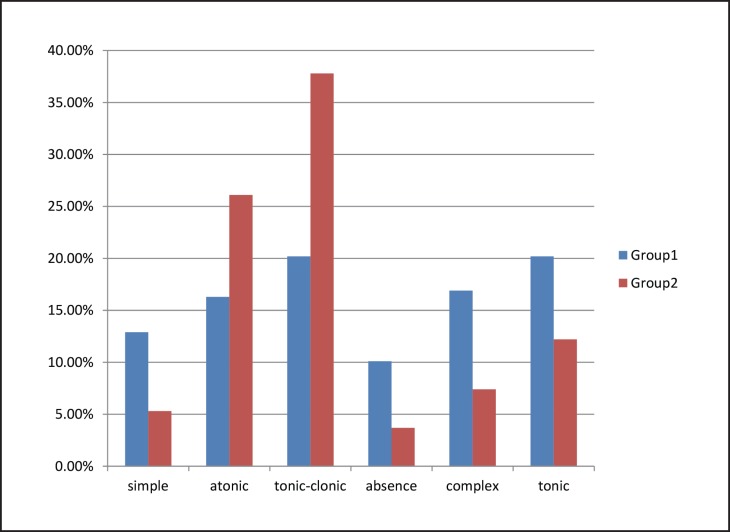
Frequency of each type of seizure in both groups

The patients’ age ranged from 1 to 15 yr; the patients ‘mean age was 8.73 yr, with mean 8.17 yr in refractory group and 9.27 yr in controls. Results showed significant relationship between seizure in first year of life and resistance to AEDs (P= 0.002). The diagnosed seizure types were simple (9.0%), atonic (21.3%), tonic-clonic (29.2%), absence (6.8%), complex (12.0%) and tonic (21.6%). As Figure 1 shows, there was a significant relationship(P= 0.000) between the type of seizure and contingency of refractory epilepsy in children. However, in both groups tonicclonic was the most common type but atonic (49/188: 26.1%) in controls while tonic (36/178: 20.2%) were second incident types. 

**Risk factors and predictors**

The present study determined the impression of independent predictors of refractory epilepsy such as; sex and age of patients, family history of seizure, type of seizure, abnormalities in EEG and imaging of the patients, other neurologic and metabolic disorders and serum drug level as a main predictor of refractory epilepsy. Table1 shows the comparison of refractory and well-controlled groups in measured prognostic factors. Prevalence of refractory epilepsy was higher in patients with other concomitant neurological disorders (59/178: 33.1%) compared to those without such disorders (12/188: 6.4%). 29.8% of patients of the resistant group had positive family history of seizure while those were 22.3% in controls. Abnormal brain imaging was seen in 12.3% of all patients but24.7% of the patients in the refractory group had abnormalities in imaging that was significant (P=0.001).We classified EEG of the patients into three groups: normal, moderately abnormal and severely abnormal. We found no relationship between prevalence of every kind of EEGs and refractory epilepsy. Another factor measured was serum level of the drug that patients were used. Table 2 shows the prevalence of epilepsy by serum drug level. Prevalence of “low serum drug level” was higher among individuals with refractory epilepsy [21.3%] compared to control group [3.7%], and this difference was statistically significant (P=0.031). This comparison represents “low serum level” as a predictor of refractory epilepsy. These variables also were factored for multivariate analysis. Data analysis based on multivariate analysis expresses concomitant neurological disorders, brain-imaging abnormalities, type of seizure and serum drug level as the most valuable predictors of resistant seizure in children.

## Discussion

Refractory epilepsy has always been one of the challenges of treating this common disease, served as the topic for clinicians to find factors associated with this problem to treat patients or early diagnosis more accurately to help the patient, his family and the healthcare system. Most of the articles considered various factors in the patients treated with the first drug therapy period who had faced resistance (2,5,11). Type of epilepsy is where less attention is paid. We considered that by dividing patients into different groups as mentioned in the results section. The current study showed there was a significant relationship between the type of epilepsy and resistance to the treatment (Figure 1). The answer to the question of whether other patient’s neurological disorders is associated with refractory epilepsy is important because most patients do not suffer from such underlying disorders before the first seizures and therefore on the basis of high response rate to the treatment, they can be assure more than the other patients. Treatment failures in both sexes are equal but patients with abnormal brain imaging are more likely to face failure in the treatment plan. In addition, to these features related to the patient’s own characteristics, low levels of antiepileptic drug, as a separate indicator was studied, resistance to therapy in patients was evaluated and was higher in the examining group so that ‘low serum levels of antiepileptic drug’ can be introduced as a predictive factor for treatment failure. We investigated the relationship between indicators and the prevalence of refractory epilepsy and as a result abnormal imaging and associated nervous disorders have the most significant role in interfering with the treatment plan, this finding recommends comprehensive studies on the relationship between the underlying disorder, its severity, patient presentations and other parameters able to help finding treatmentresistant patients. Other medical problems do not seem to hinder directly treatment success, which is why metabolic diseases in the patients we studied had the same prevalence in both groups. In a study (11) aimed to investigate the treatment of children who failed the first period of drug therapy, 90 children who had started their first medicine were studied and were followed for about 5 yr. Of the 95 patients, 48 (50.5%) were known as idiopathic epilepsy, 30 (31.6%) as cryptogenic epilepsy and 17 (17.9%) as symptomatic epilepsy. Two mostly used drugs to treat these patients were valproic acid (43.2%) and carbamazepine (38.9%). In 31.6% of the cases, treatment failed, in 40% of patients the failure happened due to the drug side effects, 37.9% was because of reduced efficacy of drugs and 24.1% of patients both these reasons (reduced drug efficacy and drug side effects) had made the results as such. Moreover, the age at diagnosis, drug selection, the highest drug dose, the etiology of epilepsy and epileptic syndromes were the factors considered in the study but no significant correlation was found between any of the factors mentioned and treatment failure with the first drug in children treated(11). Electroencephalography does not seem to be able to predict the treatment failure because we did not observe a meaningful relationship between different EEGs and the treatment outcome, but some believe the abnormal EEG pattern can serve as a prognostic factor. A study (29) was done to investigate factors predicting drug resistance in children with epilepsy, in which 636 children were studied for 2 yr and criterion for resistance to treatment was continued epilepsy despite 18 months taking 2 drugs. There had been a significant relationship between the drug resistance and age, the number of seizures at the beginning of the disease, cause of the seizures, persistent seizures, seizures associated with fever in neonates, delayed mental and motor development, intermittent seizures in one day, abnormal EEG and/or MRI and specific epileptic syndromes. Among these factors the persistent seizures, abnormal EEG, and repeated seizures in a day can be introduced as predictive factors. Considering the results of different studies with different designs, brain imaging and taking a detailed history of other neurological diseases were recommended and with regard to unique featuresof electroencephalography like being timesaving, availability, simplicity and reasonable cost, further studies with larger sample size are recommended. In another study (30), 495 patients were studied over 2 years. The aim of this study was to investigate whether the EEG epileptic form waves in patients can be used as prognostic factor of the effectiveness of the first line drug in patients or not. The results introduced the EEG epileptic form waves as an important predictive factor for the probability of first-line drug therapy failure, but regarding the findings obtained in our investigations, we cannot surely decide whether it is reasonable to trust the EEG patterns as a prognostic index or not. Generally, factors indicating the intensity of the disease can be used as predictive factors when designing the study, in this case we used the type of epilepsy and associated neurological diseases but in a study (31) about 246 people as patients with refractory epilepsy (seizures at least once a month despite taking two antiseizure drugs) were monitored and the probability of seizure inactivation and its return in the population studied was investigated. Developmental retardation, refractory epilepsy duration and the number of drugs used in the failed therapeutic process have been suggested as negative factors predicting the inactivation of the seizure, applying these topics such as developmental retardation and the number of drugs used is an evidence to verify this fact. Patients with seizure in their first year of life experienced treatment failure more, although no significant difference was observed between the mean age of the people studied in the groups but the patient’s age at diagnosis can act like a prognostic factor as a study (32) investigated the proportion of the drug therapy failure in childhood epilepsy in newly diagnosed children with epilepsy. In this study 225 patients were followed for about 4.2 yr. The results suggested approximately 32% of the patients failed the treatment with the first drug, as well as age at diagnosis, the cause of epilepsy and the type of drugs were introduced as factors associated with the failure of the first drug of choice.

**Table 1:** Demographic Features of Patients in Each Group

**Prognostic factor**		**Group1** **n: 188**	**Group2** **n: 178**	***P*** **-value**
**Gender**	Male	95[53.4]	114[60.6]	0.578
	Female	83[46.6]	74[39.4%]	
**Low serum drug level**		38[21.3]	7[3.7%]	0.031
**neurological disorder**		59[33.1]	12[6.4]	0.001
**Positive family history**		53[29.8]	42[22.3]	0.008
**Abnormal brain imaging**		44[24.7]	1[5]	0.001
**Metabolic disorder**		12[6.7]	14[7.4]	0.301
**Mean duration of seizure (min)**		4.81 min	3.87 min	0.774

**Table 2.** Group1: Patients with Refractory Epilepsy, Group 2: Well Controlled Seizures Patients

			**Level**		**Total**
			**Normal**	**Abnormal**	
**Group**	**1**	Count	140	38	178
		% within Group	78.7%	21.3%	100.0%
	**2**	Count	181	7	188
		% within Group	96.3%	3.7%	100.0%
**Total**		Count	321	45	366
		% within Group	87.7%	12.3%	100.0%

**In conclusion**, some children with seizure who suffer from refractory epilepsy need more attention and exact observation by the medical staff. Regarding this prevalent condition, it is important for the patient family to be aware of the condition. It is so important for the health system to evaluate the situation and reduce the burden of disease. There are factors able to predict whether a child will be facing treatment failure as low serum drugs level, other neurological disorders at the same time, positive family history and abnormal brain imaging. Better strategies are needed to control epilepsy and to reduce mental, social and financial pressures of refractory epilepsy on society, patients, families and healthcare systems.

## Authors’ Contribution

Dr. Mohammad Mehdi Taghdiri: Study concept and design, Development of original idea, Writing the manuscript. Mahmoud Omidbeigi: Development of original idea, Edition of manuscript, Collecting data and Statistical analysis. Dr. Sina Asaadi: Development of original idea, writing the manuscript, Collecting data and Statistical analysis, Help in writing of manuscript and edited it. Mahdi Mohebbi: Writing the manuscript, Edition of manuscript collecting data . Dr. Eznollah Azargashb: Development of original idea, Statistical analysis. Dr. Mohammad Ghofrani: Study concept and design, Development of original idea, Help in writing of manuscript and edited it. All authors agreed to be accountable for all aspects of the study including the integrity and content. and Statistical analysis consulting in the field of dyslexia and data collection. 

## References

[1] Kozyrskyj AL, Prasad AN (2004). The burden of seizures in Manitoba children: a population-based study. Can J Neurol Sci.

[2] Camfield PR, Camfield CS, Gordon Kandet al (1997). If a first antiepileptic drug fails to control a child’s epilepsy, what are the chances of success with the next drug?. J Pediatr.

[3] Arts WF, Brouwer OF, Peters ACet al (2004). Course and prognosis of childhood epilepsy: 5-year follow-up of the Dutch study of epilepsy in childhood. Brain.

[4] Berg AT, Shinnar S, Levy SR (2001). Early Development of intractable epilepsy in children: a prospective study. Neurology.

[5] Berg AT, Vickrey BG, Testa FM (2006). How long does it take for epilepsy to become intractable? A prospective investigation. Ann Neurol.

[6] Kwan P, Brodie M (2000). Early identification of refractory epilepsy. N Eng J Med.

[7] Mohanraj R, Brodie MJ (2006). Diagnosing refractory epilepsy: response to sequential treatment schedules. Eur J Neurol.

[8] Berg A (2009). Identification of Pharmacoresistant Epilepsy. Neurol Clin.

[9] Luciano AL, Shorvon SD (2007). Results of treatment changes in patients with apparently drug-resistant chronic epilepsy. Ann Neurol.

[10] Carpay HA, Arts WF, GeertsAT (1998). Epilepsy in childhood: An audit of clinical practice. Arch Neurol.

[11] Dudley RW, Penney SJ, Buckley DJ (2009). First-drug treatment failures in children newly diagnosed with epilepsy. Pediatr Neurol.

[12] Berg AT, Vickrey BG, Testa FM (2006). How long does it take epilepsy to become intractable? A prospective investigation. Ann Neurol.

[13] Spooner CG, Berkovic SF, Mitchell LA (2006). New onset temporal lobe epilepsy in children: lesion on MRI predicts poor seizure outcome. Neurology.

[14] Robinson RO, Baird G, Robinson Get al (2001). Landau– Kleffner syndrome: course and correlates with outcome. Dev Med Child Neurol.

[15] Berg AT, Shinnar S, Levy SR (2001). Defining early seizure outcomesin pediatric epilepsy: the good, the bad and the in-between. Epilepsy Res.

[16] Shinnar S, Berg AT (2009). Does antiepileptic drug therapy prevent the development of ‘‘chronic’’ epilepsy? Epilepsia 1996;37:701-8. Neurol Clin.

[17] Engel J (2004). The goal of epilepsy therapy: no seizures, no side effects,as soon as possible. CNS Spectrums.

[18] Mathern GW, Pretorius JK, Babb TL (1995). Influence of the type ofinitial precipitating injury and at what age it occurs on courseand outcome in patients with temporal lobe seizures. J Neurosurg.

[19] Cross JH, Jaykar P, Nordli D (2006). Propose criteria for referraland evaluation of children for epilepsy surgery: recommendations of the Subcomission for Pediatric Epilepsy Surgery. Epilepsia.

[20] Weiner HL, Carlson C, Ridgway EBet al (2006). Epilepsy surgery inyoung children with tuberous sclerosis: results of a novel approach. Pediatrics.

[21] Del Felice A, Beghi E, Boero G, La Neve A, Bogliun G, De Palo A (2010). Early versus late remission in a cohort of patients with newly diagnosed epilepsy. Epilepsia.

[22] Levy SR, Novotny EJ, Shinnar S (1996). Predictors of intractable epilepsy in childhood: a case–control study. Epilepsia.

[23] Berg AT, Shinnar S, Levy SR, Smith- Rappaport S, Beckerman B (2001). Early development of intractable epilepsy in children: a prospective study. Neurology.

[24] Casetta I, Granieri E, Monetti VC et al (1999). Early predictors of intractability in childhood epilepsy: a community-based case–control study in Copparo, Italy. Acta Neurologica Scandinavica.

[25] Chawla S, Aneja S, Kashyap Ret al (2002). Etiology and clinical predictors ofintractable epilepsy. Pediatric Neurology.

[26] Ko TS, Holmes GL (1999). EEG and clinical predictors of medically intractable childhood epilepsy. Clin Neurophysiol.

[27] Kwong KL, Sung WY, Wong SN (2003). Early predictors of medical intractability in childhood epilepsy. Pediatr Neurol.

[28] Oskoui M, Webster RI, Zhang X (2005). Factors predictive of outcome inchildhood epilepsy. J Child Neurol.

[29] Seker Yilmaz B, Okuyaz C, Komur M (2013). Predictors of Intractable Childhood Epilepsy. Pediatr Neurol.

[30] Kim S, Park K, Kim S, Kwon O, No S (2010). Presence of epileptiform discharges on initial EEGs are associated with failure of retention on first antiepileptic drug in newly diagnosed cryptogenic partial epilepsy: A 2-year observational study. Seizure.

[31] Callaghan B, Anand K, Hesdorffer D, Hauser W, French J (2007). Likelihood of seizure remission in an adult population with refractory epilepsy. Ann Neurol.

[32] Arhan E, Serdaroglu A, Kurt A, Aslanyavrusu M (2010). Drug treatment failures and effectivity in children with newly diagnosed epilepsy. Seizure.

